# Single-port plus one in pediatric robotic-assisted Lich-Gregoir ureteral reimplantation for vesicoureteral reflux, a comparative analysis with short-term outcomes

**DOI:** 10.1186/s12894-024-01467-y

**Published:** 2024-04-08

**Authors:** Jianglong Chen, Yingquan Kang, Shan Lin, Shaohua He, Yufeng He, Xinru Xu, Huihuang Xu, Guangxu You, Di Xu

**Affiliations:** 1 Department of Pediatric Surgery, Pediatric Medical Center, Fujian Provincial Hospital, 134 Dongjie Road, Gulou District, Fuzhou, Fujian Province China 350001; 2 Shengli Clinical Medical College of Fujian Medical University, 134 Dongjie Road, Gulou District, Fuzhou, Fujian Province China

**Keywords:** Single-port plus one, Robotic, Lich-Gregoir ureteral reimplantation, Vesicoureteral reflux, Pediatric

## Abstract

**Objective:**

To observe the safety and short-term outcomes of a new way of laparoscopic trocar placement in pediatric robotic-assisted Lich-Gregoir ureteral reimplantation for vesicoureteral reflux.

**Methods:**

The retrospective study included 32 patients under 14 years diagnosed with primary vesicoureteral reflux (VUR). All these patients underwent robotic-assisted Lich-Gregoir ureteral reimplantation in our department from December 2020 to August 2022. These patients were divided into the following groups according to the different ways of trocar placement: 13 patients in group single-port plus one (SR) and 19 patients in group multiple-port (MR). Patients’ characteristics as well as their perioperative and follow-up data were collected and evaluated.

**Results:**

There was no significant difference in the data regarding patients’ characteristics and preoperative data. These data included the grade of vesicoureteral reflux according to the voiding cystourethrogram (VCUG), and the differential degree of renal function (DRF) at the following time points: preoperative, postoperative, and comparison of preoperative and postoperative. There was no difference between the two groups. During surgery, the time of artificial pneumoperitoneum establishment, ureteral reimplantation time, and total operative time in the SR group were longer than those in the MR group. Yet only the time of artificial pneumoperitoneum establishment shows a statistical difference (*P* < 0.0001). Also, the peri-operative data, including the volume of blood loss, fasting time, hospitalization, and length of time that a ureteral catheter remained in place, and the number of postoperative complications demonstrate no difference. In addition, the SFU grade and VCUG grade at the following time point also show no difference between the two groups.

**Conclusion:**

The study demonstrates that SR in robotic-assisted Lich-Gregoir ureteral reimplantation has reached the same surgical effects as MR. In addition, the single-port plus one trocar placement receives a higher cosmetic satisfaction score from parents and did not increase the surgical time and complexity.

## Introduction

Vesicoureteral reflux (VUR) is the most common congenital anomaly of the urinary tract in children, which occurs in 1-2% of children [1]. And open surgery is traditionally accepted as the gold standard in the treatment of VUR. The effects of intravesical and extravesical approaches are similar [2]. However, open surgery is traumatic and has a long learning curve.

In recent years, minimally invasive surgery (MIS) is an increasingly important concept in both laparoscopic and Davinci-assisted surgery. Especially in pediatric surgery, reducing surgical trauma is conducive to the recovery of children after surgery. Reducing the number of trocars is a way of MIS. Single-port technology has been widely studied in recent years and widely used in various surgeries. Its usage in pediatrics is still controversial. In this study, we conducted a single-port plus one trocar placement way in pediatric robotic-assisted Lich-Gregoir ureteral reimplantation, to observe the safety and short-term effects of this new trocar placement way.

## Methods

### Patients and design

This retrospective study contains 33 patients from the Department of Pediatric Surgery at Fujian Provincial Hospital. All these patients were diagnosed with VUR and received the robotic-assisted Lich-Gregoir ureteral reimplantation. Twenty patients received a single port plus one robotic-assisted Lich-Gregoir ureteral reimplantation (SR) and thirteen patients received multiple ports robotic-assisted Lich-Gregoir ureteral reimplantation (MR). One patient in the SR group was lost to follow-up and was excluded from the study. All surgeries were done by the same team. The study was approved by the Institutional Review Board of Fujian Provincial Hospital (Approval No: K2022-07-008).

### Surgical approaches

**Fig. 1:**
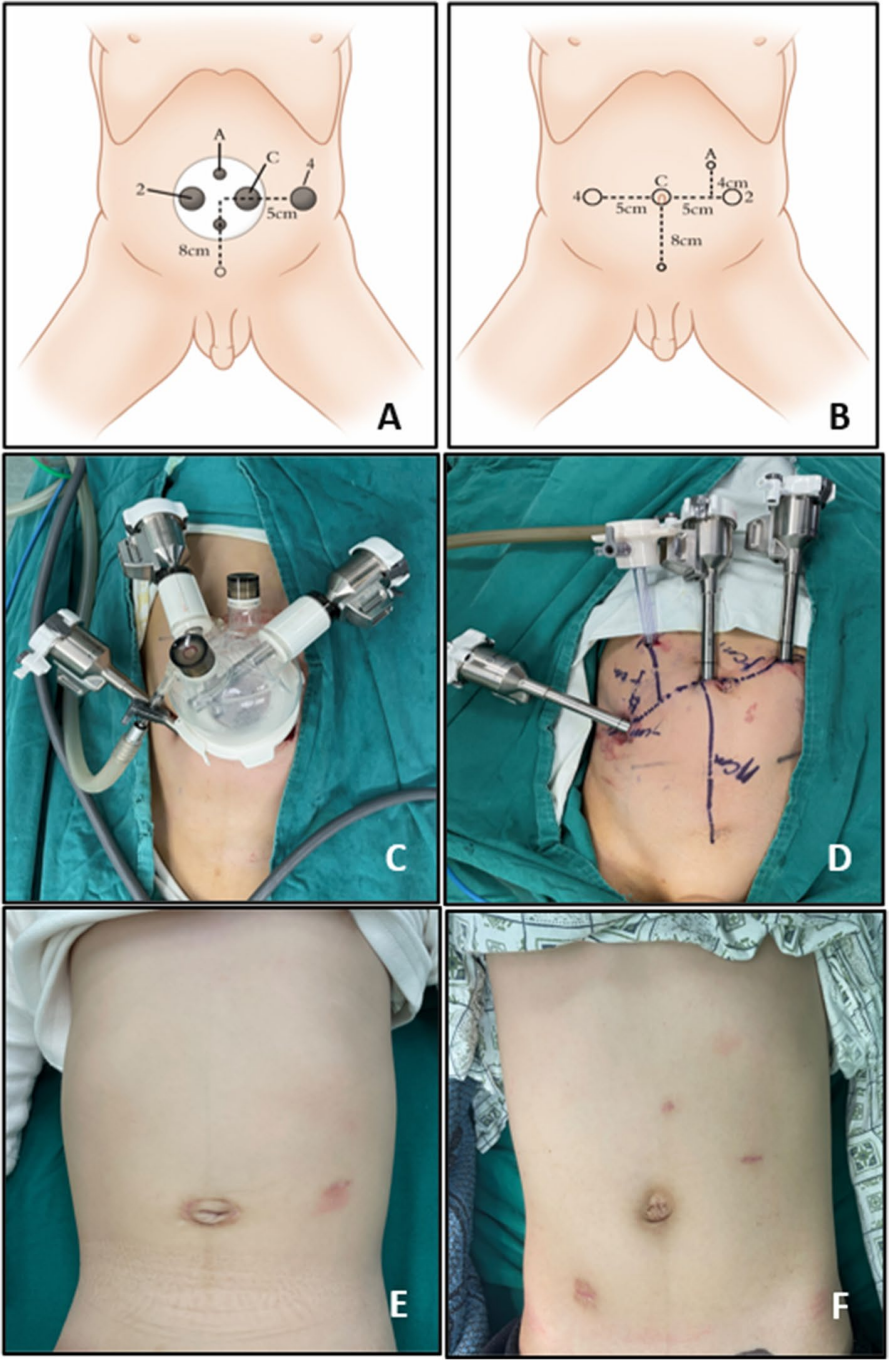
The trocar placement and postoperative cosmetic outcome of SR and MR groups. **A**, **B** the diagram of trocar placement in SR and MR groups, **C**, **D** the trocar placement in SR and MR groups, **E**, **F** cosmetic outcome of SR and MR groups

The da Vinci Xi robotic surgery system (Da Vinci, Mountain View, CA, USA) was applied to all patients in the study. For both SR and MR groups, the double J tube was retrogradely placed on the surgical ureteral side. A 25- to 30 mm arc incision was made for the SR group to insert the single port. After the artificial pneumoperitoneum was established, another 8-mm robotic trocar was inserted, which was 4–5 cm beside the single port, depending on the surgical side. Only 3 robotic arms were used, and robotic 8 mm trocars were placed in passages 2, C, and 4. Passages 2 and 4 were the operative arms of the robot and passage C was the camera. The remaining smaller passages could be used as the assistant passage (Fig. 1A, C). For the MR group, 3 robotic 8 mm trocars were placed separately on the same line or diagonal line. Another 5 mm trocar was placed on the surgical side above the connection between the two robotic trocars (Fig. 1B, D). The cosmetic outcomes at follow-up time point of SR and MR groups was shown in (Fig. 1E, F).

**Fig. 2:**
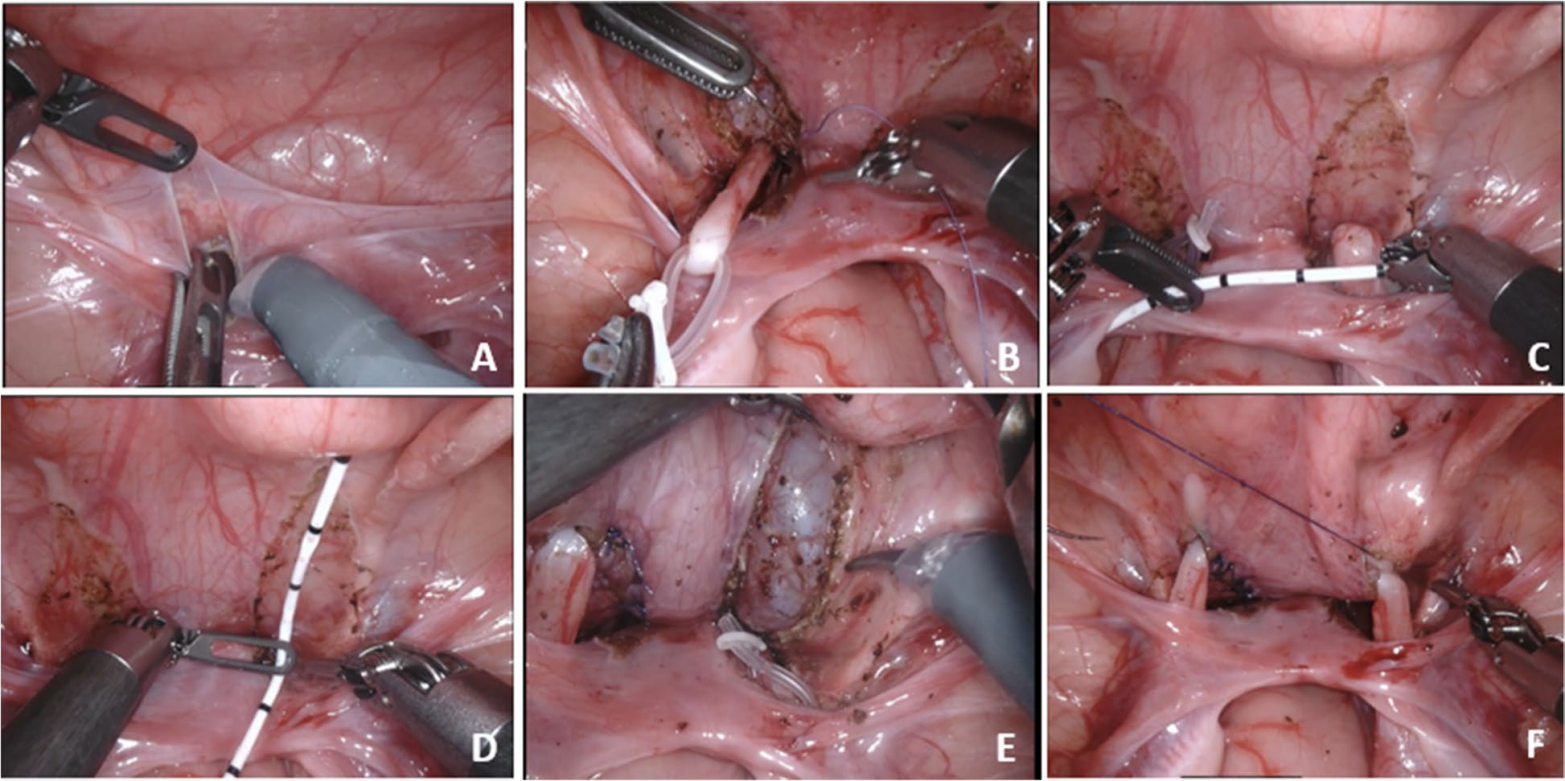
The procedures of ureteral reimplantation. **A** The refolding of the peritoneum serves as an anatomical marker to open the peritoneum and separate the ureter, **B** The hose acts as a movable suspension of the ureter, **C**, **D** Measure the diameter of the ureter and the length of the muscular layer of the bladder that needs to be incised, **E**, **F** The muscular layer of the bladder is cut until the mucosa is fully exposed, and the ureter is embedded in the muscular layer of the bladder

The following surgical steps were similar in both groups. The patient was placed in a supine position and secured to the table across the legs, shoulders, and head by silk tape. Then the Trendelenburg position (30 degrees) was set to allow the contents of the abdominal to move away from the surgical area. After all, robot docking was done and robotic instruments were installed. A transabdominal hitch provided traction and facilitated exposure to the operative field. Then the ureter of the vesicoureteral reflux side was separated (Fig. 2A, B). Cut the serous layer of the bladder and the entire muscular layer, exposing the mucosal layer. The length of the musculostomy of the bladder is 4 times the diameter of the ureter (Fig. 2C, D, E). The ureter is embedded in the muscular layer of the bladder. And then the muscular layer of the bladder was resutured (Fig. 2F). A draining tube was placed in the pelvic cavity if bladder mucosal was ruptured during the operation.

### Patient postoperative management

The urinary catheter was removed within 3 days if the mucosal was not ruptured during the operation, or it would be contained for about 7 days. A liquid diet was initiated only gastrointestinal peristalsis is restored. A diuretic nephrogram, renal static imaging, and magnetic resonance imaging were planned for 6 or 12 months after surgery, and an ultrasound of the urinary system was planned for 1, 6, and 12 months after surgery. The double J tube was removed 1 month after surgery. An ultrasound of the urinary system and urinalysis were applied whenever there is an unexplained fever or urinary tract infection.

### Data collection

Patient characteristics that were evaluated included age, gender, high, and weight. Perioperative data were evaluated, including surgical side, differential renal function (DRF), and VCUG grade. Operative data were recorded, including surgical site, whole operative time (skin to skin), trocar insertion time, Ureteral reimplantation time, blood loss, transfusions, and volumes. Postoperative and follow-up data were collected, including duration of fasting, duration of JJ tube and gastric tube use, duration of the ureteral catheter, postoperative SFU, change of pre-postoperative SFU, postoperative DRF, change of pre-postoperative DRF, postoperative APDRPU, change of pre-postoperative APDRPU, length of hospital stay, complications (urine leakage, infection, anastomotic stenosis), parents’ satisfaction scores to the surgical scar (Stony Brook Scar Evaluation Scale, SBSES) [3], and outcomes (3 months after surgery).

### Statistical analysis

The study presents values as median (range) when continuous variables do not conform to the normal distribution; otherwise, mean ± standard deviation (SD) is employed. Categorical variables are presented as numbers. Analyses were performed by SPSS (SPSS, statistics, version 21.0, IBM Corp., New York City, NY, USA), categorical variables and nonnormal distribution continuous variables were evaluated by nonparametric analysis (chi-square or Mann–Whitney H test) and normal distribution continuous variables were evaluated by Student’s test. A value of *P* < 0.05 was considered statistically significant.

## Results

A total of 32 patients were included in this study, 13 in the SR group and 19 in the MR group. There are 11 unilateral and 2 bilateral UR in the SR group, and these numbers are 13 and 6 in the MR group. There is no difference in characteristic data, including gender, height at surgery, weight at the surgical side, weight at surgery, and the following time. However, the age at surgery (*P*<0.001) and height at surgery (*P*<0.001) show statistical differences (Table 1). In addition, the number of pre-operative VCUG grades IV and V in the SR group and MR group are 13 (grade IV: 6 and grade V:7) and 19 (grade IV: 13 and grade V:6), respectively. However, pre-operative VCUG grade and pre-operative DRF show no difference between SR and MR groups (Table 1).

**Table 1:** Patients’ characteristics and preoperative data of VUR

	SR	MR	*P*
Age at surgery Median (range, month)	39.0 (4.0, 137.0)	45.0 (9.0, 95.0)	<0.001
Gender			
Male	10	11	0.45
Female	3	8	
Height at surgery	84.0 (60.0, 148.0)	104.0 (67.0, 132.0)	<0.001
Weight at surgery	13.0 (6.30, 42.0)	18.0 (5.6, 24.0)	0.80
Surgical side			0.62
Left	8	10	
Right	3	3	
Bilateral	2	6	
Pre-operative VCUG grade			0.36
III	2	3	
IV	6	13	
V	5	3	
Pre-operative DRF	36.68 ± 10.25	34.85 ± 10.76	0.64
Following time, median (month, range)	9 (6, 12)	7 (6, 16)	0.88

The time for artificial pneumoperitoneum establishment is longer in the SR group than that in the MR group (*P*<0.001) (Table 2). The time for ureteral reimplantation and the total operative time in the SR group is longer than that in the MR group both for the unilateral and bilateral sides, yet all comparisons show no difference (Table 2). The time for ureteral reimplantation is the major step of the surgery, however, the ratio of ureteral reimplantation time to total operative time also shows no difference between the two groups. In addition, there is no difference in blood loss volume and the number of transfusions between the two groups (Table 2).

**Table 2:** Patients’ intraoperative data

	SR	MR	* P *
Time of APE	11.73 ± 1.90	7.93 ± 1.14	<0.001
Ureteral reimplantation time for unilateral	100.27 ± 34.16	93.54 ± 25.71	0.59
Ureteral reimplantation time for bilateral	125.50 ± 2.12	116.0 ± 6.16	0.087
Total operative time for unilateral	164.64 ± 33.19	154.85 ± 35.94	0.50
Total operative time for bilateral	180.50 ± 2.12	169.83 ± 14.20	0.35
Ratio of UT/TT for unilateral	0.60 ± 0.10	0.61 ± 0.11	0.88
Ratio of UT/TT for bilateral	0.70 ± 0.036	0.69 ± 0.066	0.87
Blood loss volume	5.30 ± 2.72	5.42 ± 2.80	0.91
Transfusion			1.00
YES	0	0	
No	13	19	
The number converse to multiport or open surgery	0	0	1.00

*APE* Artificial pneumoperitoneum establishment, *UT* Ureteral reimplantation time, *TT* Total operative time

**Fig. 3:**
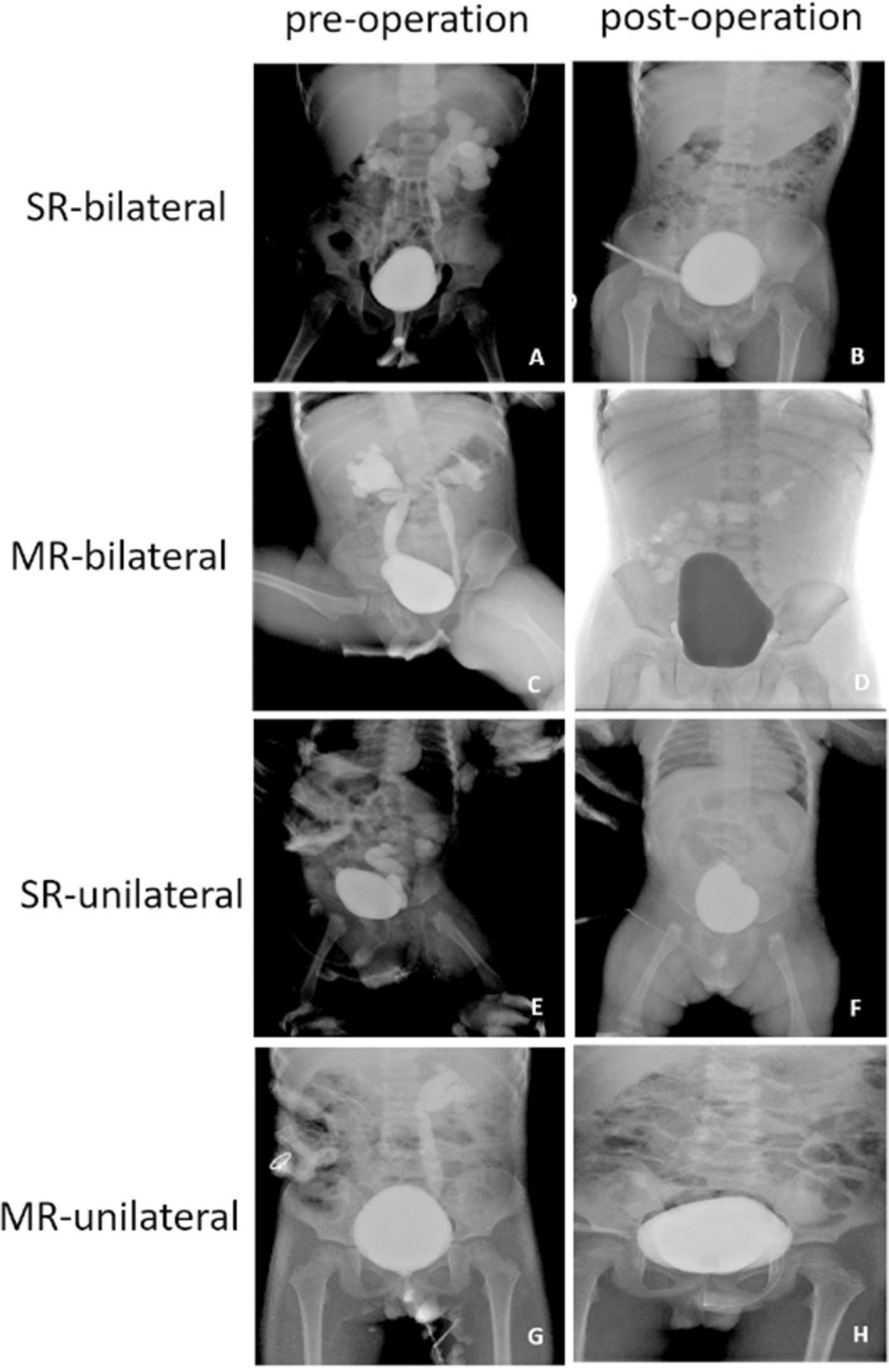
VCUG of SR and MR pre- and post-operation. **A**, **B** Pre- and post-operative VCUG of bilateral VUR in SR group, **C**, **D** Pre- and post-operative VCUG of bilateral VUR in MR group, **E**, **F** Pre- and post-operative VCUG of unilateral VUR in SR group, **G**,** H **Pre- and post-operative VCUG of unilateral VUR in SR group

There is no difference in fasting time, ureteral catheter-containing time, and hospitalization time between the SR group and MR group after the operation (Table 3). The surgery yielded good results, and the level of vesicoureteral reflux decreased significantly. The number of post-operative VCUG grade 0 in the SR group and MR group are 10 and 15, respectively (Table 3). The VCUG of SR and MR pre- and post-operation in both bilateral and unilateral are showed in Fig. 3. Thought 3 and 4 patients remain a VUR grade of I or II in the SR group and MR group, respectively (Table 3). The pictures of VCUG in different groups at different time points are shown in Fig. 3. The post-operative DRF in the SR group has increased from 36.68 ± 10.25 to 44.57 ± 6.48, and that is from 34.85 ± 10.76 to 43.01 ± 7.87 in the MR group (Table 3). The complications of ureteral reimplantation include bladder leakage, anastomotic stenosis, and urinary tract infection. However, one patient in each group happens to have bladder leakage and the rests develop no other complications (Table 3).

**Table 3:** Patients’ postoperative and follow-up data

	SR	MR	*P*
Fasting time	1.31 ± 0.48	1.42 ± 0.61	0.58
Ureteral catheter containing time	4.15 ± 0.69	4.47 ± 1.35	0.44
Hospitalization time	9.77 ± 2.13	10.47 ± 1.87	0.33
Post-operative VCUG grade			1.00
0	10	15	
I	2	2	
II	1	2	
III	0	0	
IV	0	0	
V	0	0	
Post-operative DRF	44.57 ± 6.48	43.01 ± 7.87	0.56
Complication(YES/NO)	1/13	1/19	1.00
Bladder leakage(YES/NO)	1/30	1/19	1.00
Anastomotic stenosis (YES/NO)	0/13	0/19	1.00
Urinary tract infection (YES/NO)	0/13	0/19	1.00
SBSES scores of surgical scar	4.00 ± 0.71	3.37 ± 0.76	0.024

*VCUG* voiding cystourethrogram, *SBSES* Stony Brook Scar Evaluation Scale

## Discussion

Here we demonstrate single-port plus one in pediatric robotic-assisted Lich-Gregoir ureteral reimplantation for vesicoureteral reflux for the first time. The result shows high safety and good surgical effects.

Robotic-assisted or laparoscopic single-port surgery is wildly applied in various adult subspecialty [3–6]. And robotic-assisted single-port surgery is successfully used in pediatric pyeloplasty and cholecystectomy [7, 8]. However, few studies focus on applying single-port or single-port plus one in pediatric Lich-Gregoir ureteral reimplantation. Therefore, it is important to investigate the safety and outcomes of this technology. The advantages of robotic-assisted surgery include the filtration of tremors, improved precision and dexterity, and three-dimensional high-definition vision [9]. Which leads to fewer post-operative complications and shorter hospitalization time. The hospitalization time of the SR group is about the same as that of the MR group in this study. In addition, the whole time, ureteral reimplantation time and artificial pneumop, peritoneum establishment time in the SR group are longer than in the MR group. Yet, these data do not show statistical differences. And the UT/WT ratio between the two groups implies that single-port plus one technology does not increase the complexity of the surgery. All these data demonstrate that single-port plus one technology does not prolong the surgery procedure or increase post-operative complications. This means the safety of the SR in pediatric Lich-Gregoir ureteral reimplantation.

Conventional views that da Vinci surgery requires sufficient intracavitary space and trocars’ distance to avoid collisions with robotic arms and that an operating triangle needs to be formed so that the surgery can be performed smoothly [10–12]. The single-port application in pediatrics, especially in infants or low body weight, may lead to a difficult situation. However, single-port lead to more severe laparoscopic instrument collisions and the loss of the operating triangle. The single-port plus one solves the contradiction of single port in pediatric robotic surgery, without losing the benefits. Though the da vinci SP system has been applied in pediatric surgery [8, 13], research on the single port of da vinci Xi system is still necessary, especially in pediatric patients. It must be emphasized that robotic-assisted single-port plus one operation requires rich experience in multiple-port for surgeons. In our study, all surgeries are finished by the same surgeon and the same team, and the single-port plus one technique was applicated after our team was proficient in multiple-port surgery. All previous studies have shown the learning curve in single-site surgery with multiple experience [14–16]. In addition, pediatric da Vinci surgery is quite different from that in adult surgery. First, pediatric da Vinci surgery uses only 3 robotic arms, not 4 as that in adults. Then the distance between parts could be reduced from 8 cm to 4-6 cm, for the abdominal area of the pediatric is too small to keep the 8 cm port distance. The minimum body weight in this study is only 5.6 kg. In addition, the distance between ports and target anatomy is also reduced in pediatrics but has to keep enough space to make sure the inserted robotic instrument can be opened and used. The robotic ports movement strategy in pediatrics must be a small step every time, which is also different from adults. Applying all these skills, we could use multiple ports and single ports plus one in pediatric surgery and avoid instrument clashing.

In the process of developing the single-port plus one technique, we have summarized some experience and found some more practical surgical techniques in practice. Opening the peritoneum at the reverse fold of the peritoneum allows structures such as vas deferens, ureters, etc. to be clearly identified, which can better protect them from damage. Bladder suspensions provide better exposure to the surgical area, and hanging the ureter with a thin hose avoids too much clamping on the ureter. All of these measures are designed to provide the surgeon with a stable, clear view of the procedure while minimizing the patient’s injury.

Yet the application of robot-assisted single-port technology in pediatric surgery is not mature. And the sample of this study is too small. In addition, the safety and effects of single-port or single-port plus one is comparable in several pediatric surgeries [6, 17, 18]. However, the application effect of this technology in various surgeries in pediatric surgery, especially pediatric urology, needs to be verified by more research institutions and larger population samples.

## Conclusion

The study demonstrates that SR in robotic-assisted Lich-Gregoir ureteral reimplantation is safe and has comparable short-term effects as MR. In addition, the single-port plus one trocar placement receives a higher cosmetic satisfaction score from parents and did not increase the surgical time and complexity.

## Data Availability

All data generated or analysed during this study are included in this published article, further inquiries can be available from the corresponding author on reasonable request.

## Abbreviations

VUR: Vesicoureteral reflux
SR: Single-port plus one
MR: Multiple-port
VCUG: Voiding cystourethrogram
DRF: Degree of renal function
MIS: Minimally invasive surgery
SD: Standard deviation
APE: Artificial pneumoperitoneum establishment
UT: Ureteral reimplantation time
TT: Total operative time
